# Distribution patterns and evolution of antimicrobial resistance in Gram-negative bacteria within the intensive care unit of a tertiary hospital from 2019 to 2024

**DOI:** 10.3389/fmicb.2025.1587132

**Published:** 2025-05-15

**Authors:** Xiaoying Chen, Xin Liu, Wenyan Ren, Hongyan Li, Siyun Yang

**Affiliations:** ^1^Department of Pharmacy, Nanchong Key Laboratory of Individualized Drug Therapy, Beijing Anzhen Nanchong Hospital of Capital Medical University & Nanchong Central Hospital, Nanchong, China; ^2^Department of Laboratory, Beijing Anzhen Nanchong Hospital of Capital Medical University & Nanchong Central Hospital, Nanchong, China

**Keywords:** intensive care unit (ICU), Gram-negative bacteria, antimicrobial agents, antibiotic resistance, distribution patterns

## Abstract

**Background:**

This study aims to investigate the distribution and drug resistance of Gram-negative bacteria in the intensive care unit (ICU) of a tertiary general hospital in Sichuan Province, with the goal of promoting rational antibiotic use and reducing multidrug resistance.

**Methods:**

A retrospective analysis was conducted on the distribution and drug resistance of Gram-negative bacteria in ICU samples collected from January 2019 to December 2024.

**Results:**

A total of 83,944 culture samples were analyzed, primarily blood (45.27%) and sputum (41.34%) specimens, with a steady increase in sample types annually. A total of 7,211 strains were isolated, 76.43% of which were from respiratory tract specimens. The predominant pathogens included *Klebsiella pneumoniae* (31.17%), *Acinetobacter baumannii* (30.11%), *Escherichia coli* (14.05%), and *Pseudomonas aeruginosa* (11.34%). The detection rates for *carbapenem-resistant A. baumannii* (CRAB) were 61.88%, *carbapenem-resistant K. pneumoniae* (CRKP) 29.28%, *carbapenem-resistant P. aeruginosa* (CRPA) 5.80%, and *carbapenem-resistant E. coli* (CREC) 3.04%. Susceptibility testing revealed fluctuating resistance rates for *E. coli* over the past 6 years. Notably, *K. pneumoniae* exhibited significant resistance to carbapenems (e.g., imipenem) and third-generation cephalosporins (e.g., ceftazidime).

**Conclusion:**

From 2019 to 2024, the ICU experienced a severe problem with Gram-negative drug-resistant bacteria, particularly *Enterobacteriaceae* resistant to third-generation cephalosporins. *A. baumannii* isolates demonstrated resistance to most antibiotics, underscoring the need for continuous monitoring and the selection of effective antibiotics based on clinical practice.

## 1 Introduction

Antimicrobial resistance represents one of the most significant global public health challenges. In 2019, it was estimated that this issue contributed to approximately 4.95 million deaths worldwide, with a particularly pronounced impact in low- and middle-income countries (Murray, C. J. L. et al., 2022). In May 2024, the World Health Organization (WHO) revised its list of bacterial pathogens that pose the greatest threat to human health. Notably, antibiotic-resistant Gram-negative bacteria (GNB), including *Acinetobacter baumannii* and several pathogens within the *Enterobacterales* order, were highlighted as top priorities. These bacteria are especially concerning due to their ability to transfer resistance genes, their growing resistance to current treatments, and the severity of the infections they cause, which significantly contribute to the global disease burden. GNB are a leading etiology of nosocomial infections in intensive care units (ICUs), with their epidemiological distribution and antimicrobial resistance (AMR) profiles critically influencing the formulation of evidence-based anti-infective strategies. The escalating use of broad-spectrum antibiotics has precipitated a marked increase in multidrug-resistant (MDR) and extensively drug-resistant (XDR) GNB strains, particularly in ICUs, where these pathogens pose formidable challenges to infection prevention and patient outcomes. The evolution of AMR in GNB is multifactorial. It is driven by antibiotic selection pressure, suboptimal adherence to infection control protocols, and genetic mechanisms such as horizontal gene transfer and efflux pump overexpression (Dulanto Chiang and Dekker, 2019; ICPIC, 2019). ICU patients are particularly susceptible to drug-resistant infections due to immunosuppression, critical illness, and frequent exposure to invasive devices (e.g., mechanical ventilation, central venous catheters), which disrupt innate barriers and promote microbial colonization (Jiang et al., 2023). Furthermore, The heterogeneous and prolonged use of antibiotics in ICUs accelerates the clonal expansion and dissemination of resistant strains (Doron and Davidson, 2013). This study aimed to describe the epidemiological patterns and temporal AMR trends of GNB isolates in a tertiary hospital’s ICU. By analyzing retrospective microbiological surveillance data, we aimed to inform targeted antimicrobial stewardship interventions and optimize infection control policies. Understanding the dynamic interplay between pathogen distribution, resistance mechanisms, and clinical practices is crucial for mitigating the emergence of MDR/XDR pathogens and improving therapeutic efficacy.

## 2 Methodology

### 2.1 Sources of bacterial specimens

Bacterial specimens were obtained from patients admitted to the intensive care unit (ICU) of a large, tertiary-level, general teaching hospital in Sichuan Province between January 2019 and December 2024. Gram-negative bacilli strains isolated from sputum, urine, blood, and other clinical specimens were included in the study.

### 2.2 Isolation, culture, and identification of bacteria

All specimens were collected from ICU patients under sterile conditions. Blood samples were obtained via venipuncture into pre-labeled aerobic and anaerobic culture bottles. Sputum (both aspirated and expectorated), puncture fluid, and urine were promptly transported to the laboratory upon collection. Upon arrival, blood cultures were incubated using the BACTEC FX40 system, while sputum samples underwent Gram staining and quality assessment prior to inoculation onto agar plates. Bacterial isolation and culture were performed in strict adherence to the guidelines established by the Clinical and Laboratory Standards Institute (CLSI).

### 2.3 Antimicrobial susceptibility testing

The drug sensitivity testing was conducted in accordance with the guidelines recommended by the CLSI. Antimicrobial susceptibility was assessed using both automated instrumentation and the disk diffusion method. The minimum inhibitory concentrations (MICs) of antimicrobial agents were determined utilizing the Trek system. Susceptibility, intermediate, and resistance categories were assigned based on the breakpoints specified in the CLSI’s “Performance Standards for Antimicrobial Susceptibility Testing,” 31st edition, published in 2021. Definition of drug-resistant strains are listed in Supplementary material.

### 2.4 Statistical methods

Count data were presented as n (%) and trend *p* values were calculated using GraphPad Prism 10.0 (San Diego, United States), employing the chi-square test for trend. A *p*-value of less than 0.05 was considered indicative of statistical significance.

## 3 Results

### 3.1 Trend analysis of culture sample distribution and pathogen detection from 2019 to 2024

**Figure 1:**
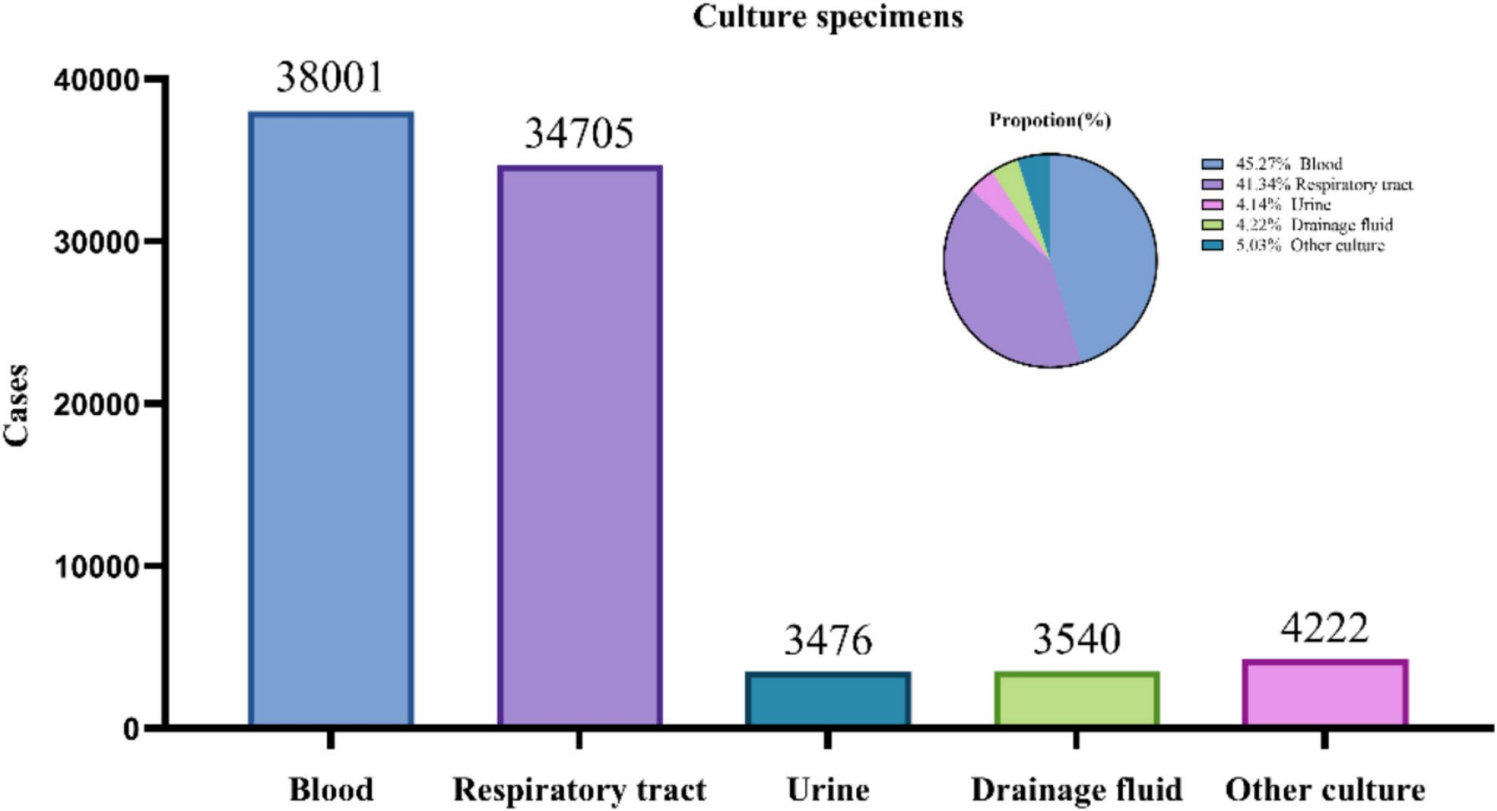
Source and proportion of total culture specimens.

From 2019 to 2024, a total of 83,944 culture samples were collected. Blood samples (38,001; 45.27%) and respiratory tract samples (33,705; 41.34%) constituted the majority of the samples. The pathogens detected in drainage cultures (3,476; 4.14%), urine cultures (3,540; 4.22%), and other types of cultures (4,222; 5.03%) are summarized in Figure 1.

**Figure 2:**
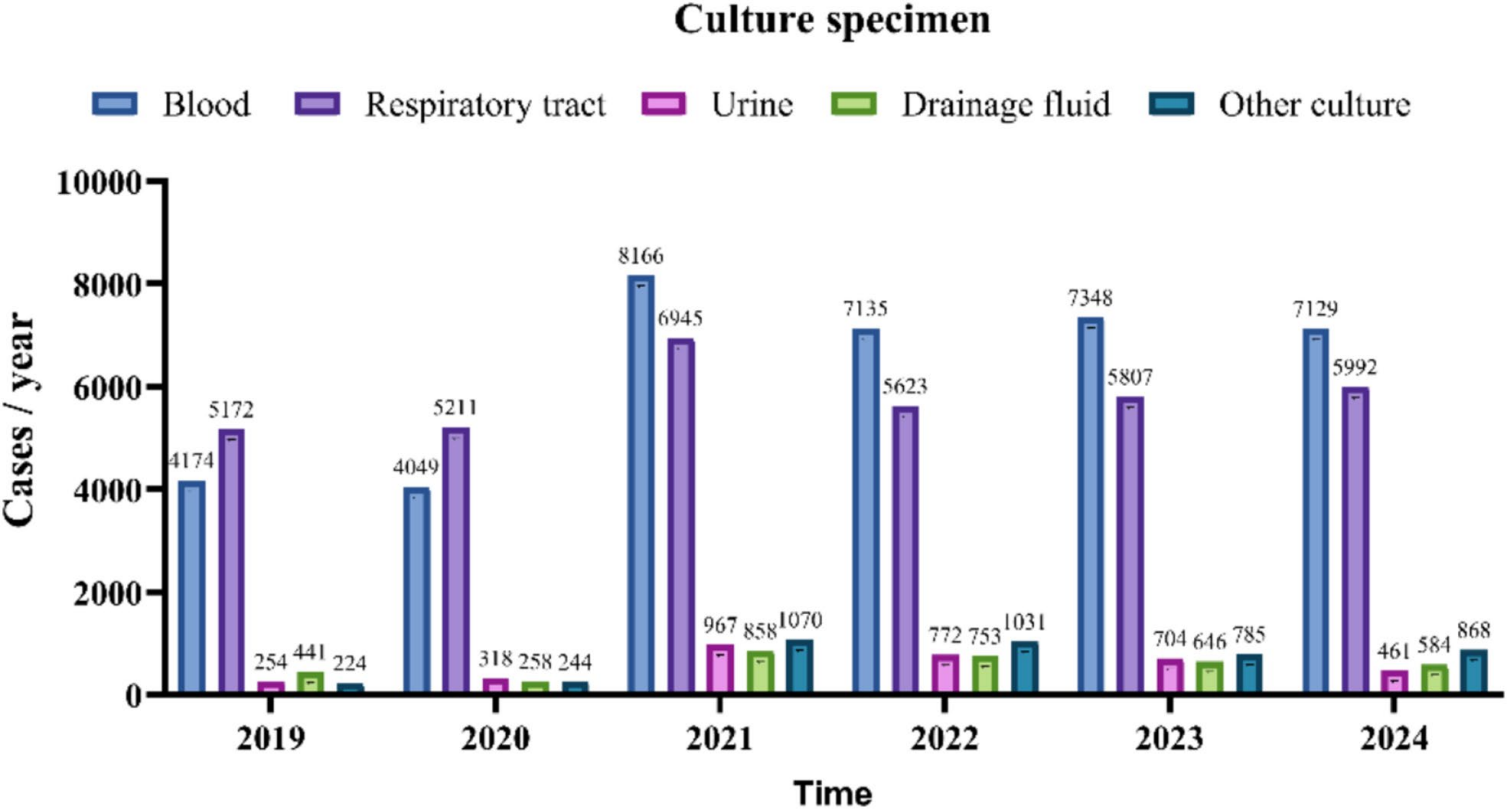
Cases of each culture specimen/year.

**Figure 3:**
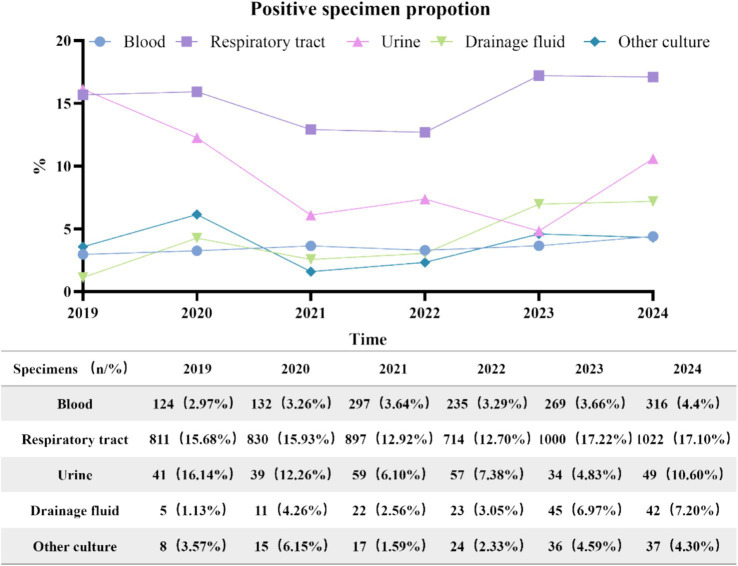
Source and distribution of positive specimens and proportion.

Between 2019 and 2024, the number of various types of culture specimens exhibited a consistent upward trend, as illustrated in Figure 2. A total of 7,211 positive specimens were cultured from 2019 to 2024. The annual distribution was as follows: 989 in 2019, 1,027 in 2020, 1,292 in 2021, 1,053 in 2022, 1,384 in 2023, and 1,466 in 2024. Specimen types included 5,274 respiratory specimens (73.1%), 1,373 blood specimens (19.0%), 148 drainage fluid specimens (2.1%), 279 urine specimens (3.9%), and 137 other types of specimens (1.9%). Respiratory and drainage fluid specimens demonstrated a progressive increase in positivity rates over the study period, while blood cultures maintained a stable proportion of positive results. In contrast, urine cultures exhibited an initial rise in positivity rates followed by a gradual decline after 2021, as illustrated in Figure 3.

### 3.2 Detection rate of Gram-negative bacteria in ICU during 2019–2024

This study examined 83,944 clinical isolates of Gram-negative bacteria collected from bloodstream, respiratory tract, urinary tract, and abdominal infections. Between 2019 and 2024, *Klebsiella pneumoniae* and the *A. baumannii complex* constituted 31.22 and 30.38% of the total cases, respectively. *K. pneumoniae* exhibited the highest overall burden, with a significant increase from 266 cases (27.01%) in 2019 to 470 cases (34.28%) in 2023, peaking that year. Although there was a slight decline to 400 cases (35.24%) in 2024, it remains a critical *pathogen*. *Haemophilus influenzae* demonstrated an alarming 11.6-fold increase from 5 cases to 56 cases, indicating emerging clinical significance. *Serratia marcescens* surged from 2 cases (0%) in 2019 to 29 cases (2.56%) in 2024, reflecting potential shifts in infection dynamics. *Pseudomonas aeruginosa* showed a sharp decline, dropping from 133 cases (13.50%) in 2019 to 61 cases (5.37%) in 2024. *A. baumannii complex* experienced significant fluctuations, peaking at 416 cases (42.23%) in 2019 before plummeting to 203 cases (19.41%) in 2022, followed by partial recovery to 314 cases (27.67%) in 2024. *Stenotrophomonas maltophilia*, *Enterobacter cloacae complex*, and *Citrobacter freundii* exhibited irregular patterns. *Klebsiella oxytoca*, *Enterobacter aerogenes*, and *Burkholderia cepacia* maintained consistently low counts. Refer to Table 1 for detailed data.

**Table 1:** Distribution and composition of pathogens in ICU: Gram-negative bacteria from 2019 to 2024 [*n* (%)].

Pathogenic Bacteria	2019 (985)	2020 (1038)	2021 (1325)	2022 (1046)	2023 (1371)	2024 (1135)	Total (7211)	*χ*^2^	*P*
*Escherichia coli*	69 (7.01)	70 (6.74)	234 (17.66)	189 (18.07)	201 (14.66)	250 (17.02)	1,013 (14.05)	108.46	<0.001
*Klebsiella pneumoniae*	266 (27.01)	306 (29.48)	384 (28.98)	328 (31.36)	470 (34.28)	494 (33.63)	2,248 (31.17)	15.55	0.008
*Acinetobacter baumannii complex*	416 (42.23)	409 (39.40)	358 (27.02)	203 (19.41)	396 (28.88)	389 (26.47)	2,171 (30.11)	95.21	<0.001
*Pseudomonas aeruginosa*	133 (13.50)	131 (12.62)	157 (11.85)	180 (17.21)	117 (8.53)	100 (6.81)	818 (11.34)	52.37	<0.001
*Haemophilus influenzae*	5 (0.51)	5 (0.48)	6 (0.54)	13 (1.24)	23 (1.68)	63 (4.29)	115 (1.59)	199.11	<0.001
*Proteus mirabilis*	26 (2.64)	28 (2.70)	30 (2.26)	30 (2.87)	35 (2.55)	30 (2.04)	179 (2.48)	1.26	0.939
*Stenotrophomonas maltophilia*	18 (1.83)	20 (1.93)	61 (4.60)	44 (4.21)	35 (2.55)	34 (2.31)	212 (2.94)	19.61	0.001
*Enterobacter cloacae complex*	37 (3.76)	36 (3.47)	29 (2.19)	28 (2.68)	46 (3.36)	58 (3.95)	234 (3.25)	6.77	0.239
*Klebsiella oxytoca*	4 (0.41)	0	3 (0.23)	1 (0.1)	10 (0.73)	5 (0.34)	23 (0.32)	16.49	0.006
*Enterobacter aerogenes*	2 (0.20)	2 (0.19)	0	1 (0.1)	0	0	5 (0.07)	–	–
*Burkholderia cepacia*	9 (0.91)	9 (0.87)	15 (1.3)	12 (1.15)	15 (1.09)	8 (0.54)	68 (0.94)	3.2	0.669
*Citrobacter freundii*	0	3 (0.29)	4 (0.30)	5 (0.48)	2 (0.15)	4 (0.27)	18 (0.25)	5.31	0.379
*Serratia marcescens*	0	3 (0.29)	31 (2.3)	8 (0.76)	17 (1.24)	34 (2.31)	93 (1.29)	64.82	<0.001
Other	0	16 (1.4)	13 (0.9)	4 (0.38)	4 (0.29)	0	37 (0.51)	52.15	<0.001

– indicates that the number of cases is too low.

#### 3.2.1 Gram-negative bacteria across various infection sites

**Figure 4:**
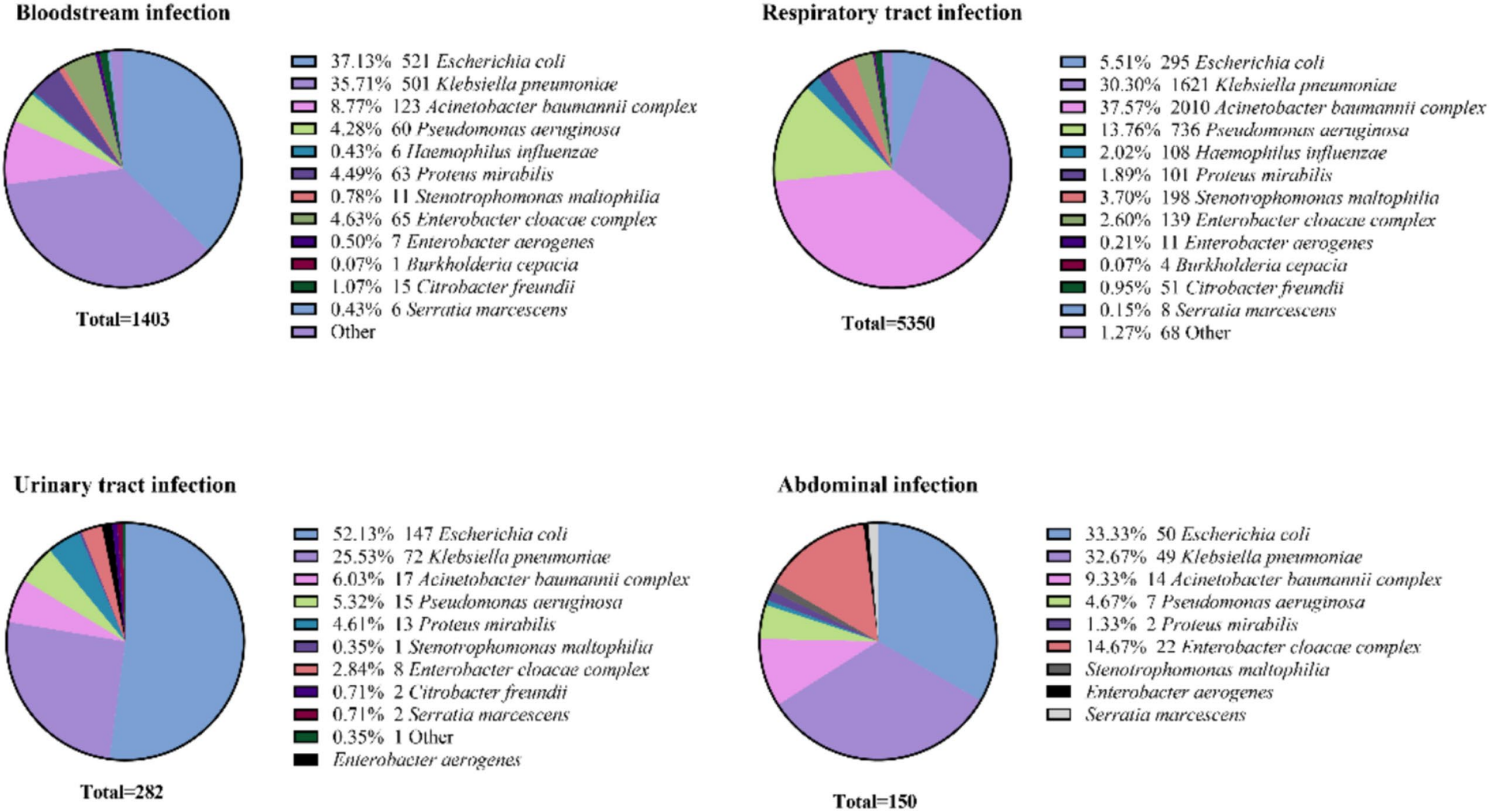
Distribution of Gram-negative bacteria at different sites of infection.

*Escherichia coli* was the predominant pathogen in urinary tract infections (52.13%, 147/295) and bloodstream infections (37.13%, 485/1403), followed by abdominal infections (33.33%, 50/150), while it was least isolation in respiratory tract infection (5.51%, 95/5350). In contrast, *K. pneumoniae* ranked second or third at all infection sites, with the highest detection rate observed in respiratory infections (30.30%, 1621/5350). *P. aeruginosa* and the *A. baumannii complex* were predominantly isolated from respiratory tract infections (13.76%, 736/5350 and 37.57%, 2010/5350, respectively), with lower frequencies in abdominal infections (4.67%, 6/150 and 9.33%, 11/150). *B. cepacia* showed moderate prevalence in bloodstream (0.07%, 15/1403) and respiratory infections (0.07%, 49/5133), as depicted in Figure 4.

#### 3.2.2 Detection rates of multidrug-resistant Gram-negative bacteria in the ICU

We analyzed multidrug-resistant Gram-negative bacteria, including *carbapenem-resistant E. coli* and *K. pneumoniae*, as well as *carbapenem-resistant P. aeruginosa* and *A. baumannii* (Table 2). The average detection rates for carbapenem-resistant *A. baumannii*, *E. coli*, *K. pneumoniae*, and *P. aeruginosa* demonstrate a pattern of fluctuation.

**Table 2:** Detection rates of multidrug-resistant Gram-negative bacteria in the ICU from 2019 to 2024 [*n* (%)].

Gram-negative bacteria	2019	2020	2021	2022	2023	2024	Total	χ^2^	*P*
*Carbapenem-resistant E. coli* (CRECO)	5 (2.22)	6 (2.54)	5 (2.08)	7 (3.33)	8 (3.54)	12 (4.33)	42 (3.04)	4.86	0.433
*Carbapenem-resistant Klebsiella pneumoniae* (CRKP)	74 (32.89)	87 (36.86)	74 (30.83)	57 (27.14)	54 (23.89)	68 (24.55)	414 (29.28)	10.78	0.056
*Carbapenem-resistant Pseudomonas aeruginosa* (CRPA)	5 (2.22)	12 (5.08)	15 (6.25)	13 (6.19)	18 (7.96)	19 (6.86)	82 (5.80)	9.32	0.097
*Carbapenem-resistant Acinetobacter baumannii* (CRAB)	141 (62.67)	131 (55.51)	146 (60.83)	133 (63.33)	146 (64.60)	178 (64.26)	875 (61.88)	9.89	0.078

### 3.3 Antimicrobial resistance profiles of *Escherichia coli* to commonly used antibiotics from 2019 to 2024

**Figure 5:**
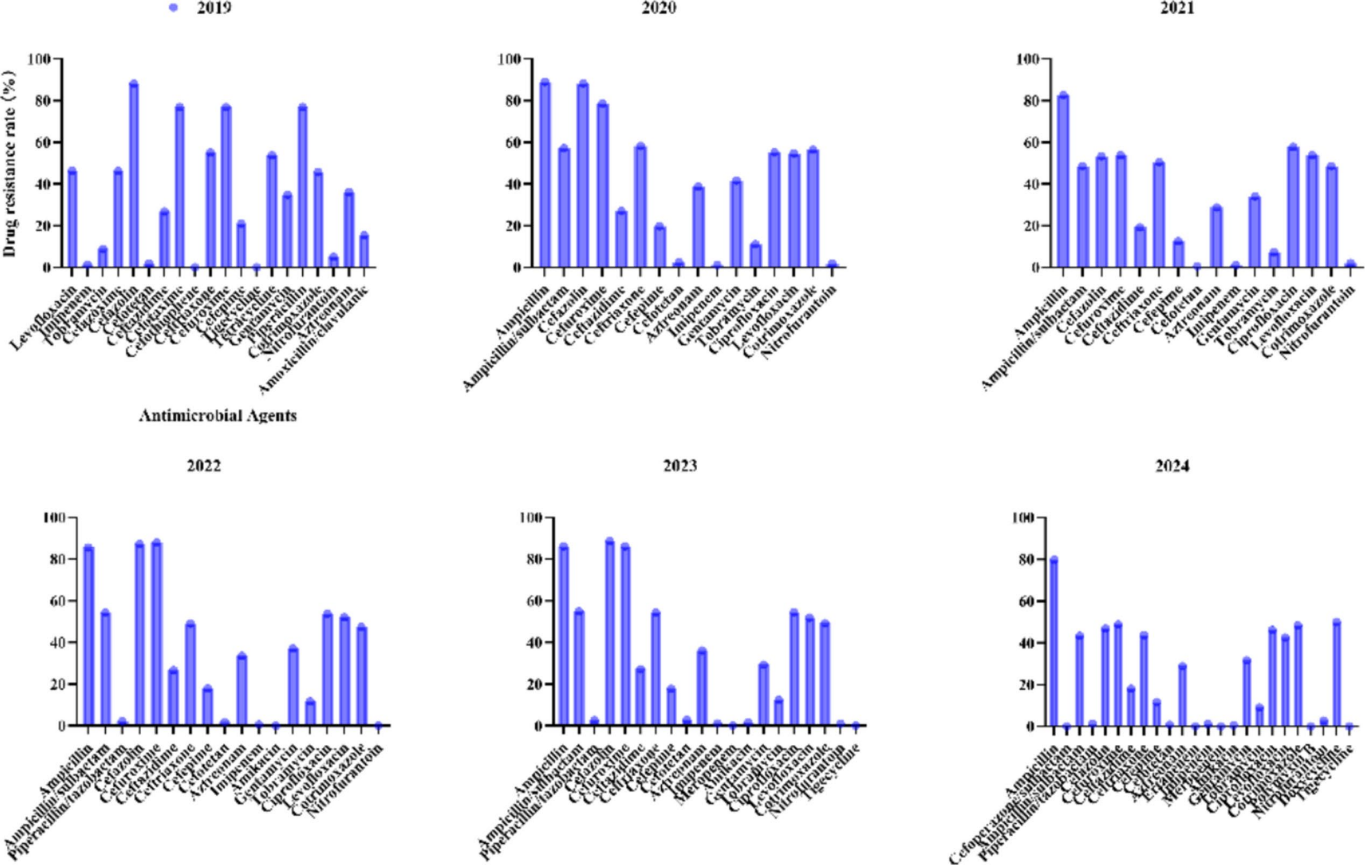
Antimicrobial resistance profiles of *Escherichia coli* to commonly used antibiotics.

For *E. coli*, recent years have witnessed significant fluctuations in antibiotic resistance patterns (Figure 5). Ampicillin has consistently exhibited high resistance levels (>79.8% annually), peaking at 89% in 2020 and marginally declining to 79.8% by 2024. Similarly, cephalosporins such as ceftriaxone showed an initial increase from 55.1% in 2019 to 54.3% in 2023, followed by a notable reduction to 43.7% in 2024, underscoring the need for continuous surveillance. Fluoroquinolones, including ciprofloxacin (from 55.2 to 46.1%) and levofloxacin (from 54.6 to 42.5%), demonstrated gradual declines between 2020 and 2024. Carbapenems (imipenem/meropenem) maintained exceptional efficacy, with resistance rates consistently ≤1.3%. Piperacillin/tazobactam exhibited minimal resistance (<2.8%), further decreasing to 1.3% by 2024. Nitrofurantoin continued to show negligible resistance (0–2.8%), highlighting its reliability for treating urinary tract infections.

### 3.4 Antimicrobial resistance profiles of *Klebsiella pneumoniae* to commonly used antibiotics from 2019 to 2024

**Figure 6:**
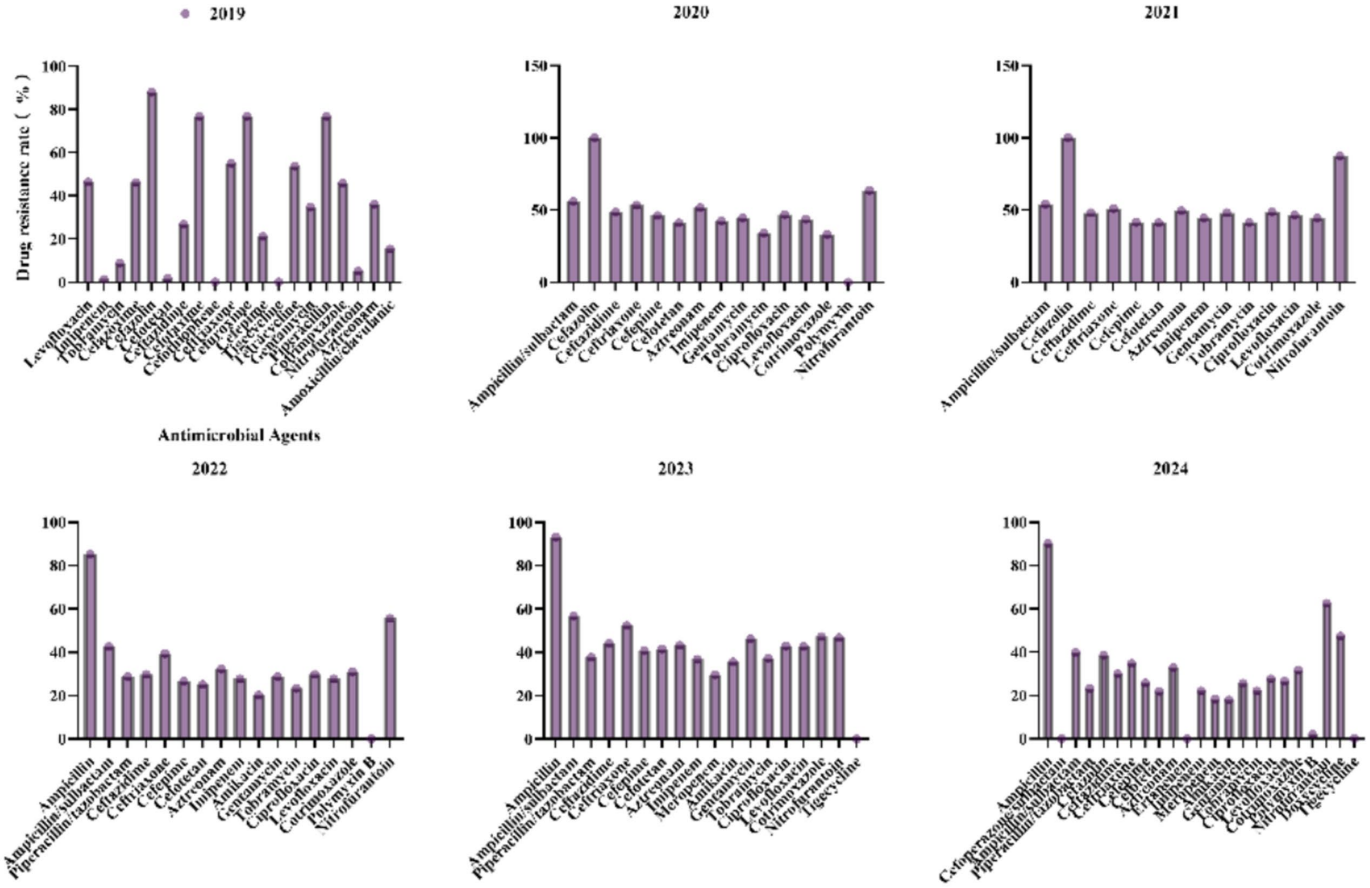
Antimicrobial resistance profiles of *Klebsiella pneumoniae* to commonly used antibiotics.

For *K. pneumoniae*, antibiotic resistance patterns have shown significant fluctuations in recent years (Figure 6). Ampicillin/Sulbactam resistance peaked at 56.9% in 2023 and then dropped to 39.8% in 2024. Ceftazidime resistance increased from 26.8% in 2019 to 48.6% in 2020, before decreasing to 29.9% in 2024. Piperacillin/Tazobactam resistance rose from 28.7% in 2022 to 37.7% in 2023, then fell to 23.1% in 2024, indicating intermittent selective pressures. Imipenem resistance sharply increased from 1.3% in 2019 to 36.8% in 2023, then declined to 22% in 2024, suggesting the emergence of carbapenemase-producing strains. Meropenem resistance was 29.7% in 2023 and decreased to 18.2% in 2024, highlighting a threat to last-line therapies. Resistance to Ciprofloxacin and Levofloxacin decreased from 46.9 and 43.8% in 2020 to 27.6 and 26.5% in 2024, likely due to reduced fluoroquinolone use. Polymyxin B and Tigecycline resistance remained low (0–2.1%), maintaining their efficacy against multidrug-resistant strains. The rise in carbapenem resistance underscores the need for robust antimicrobial stewardship programs and enhanced surveillance for carbapenemase genes.

### 3.5 Antimicrobial resistance profiles of *Pseudomonas aeruginosa* to commonly used antibiotics from 2019 to 2024

**Figure 7:**
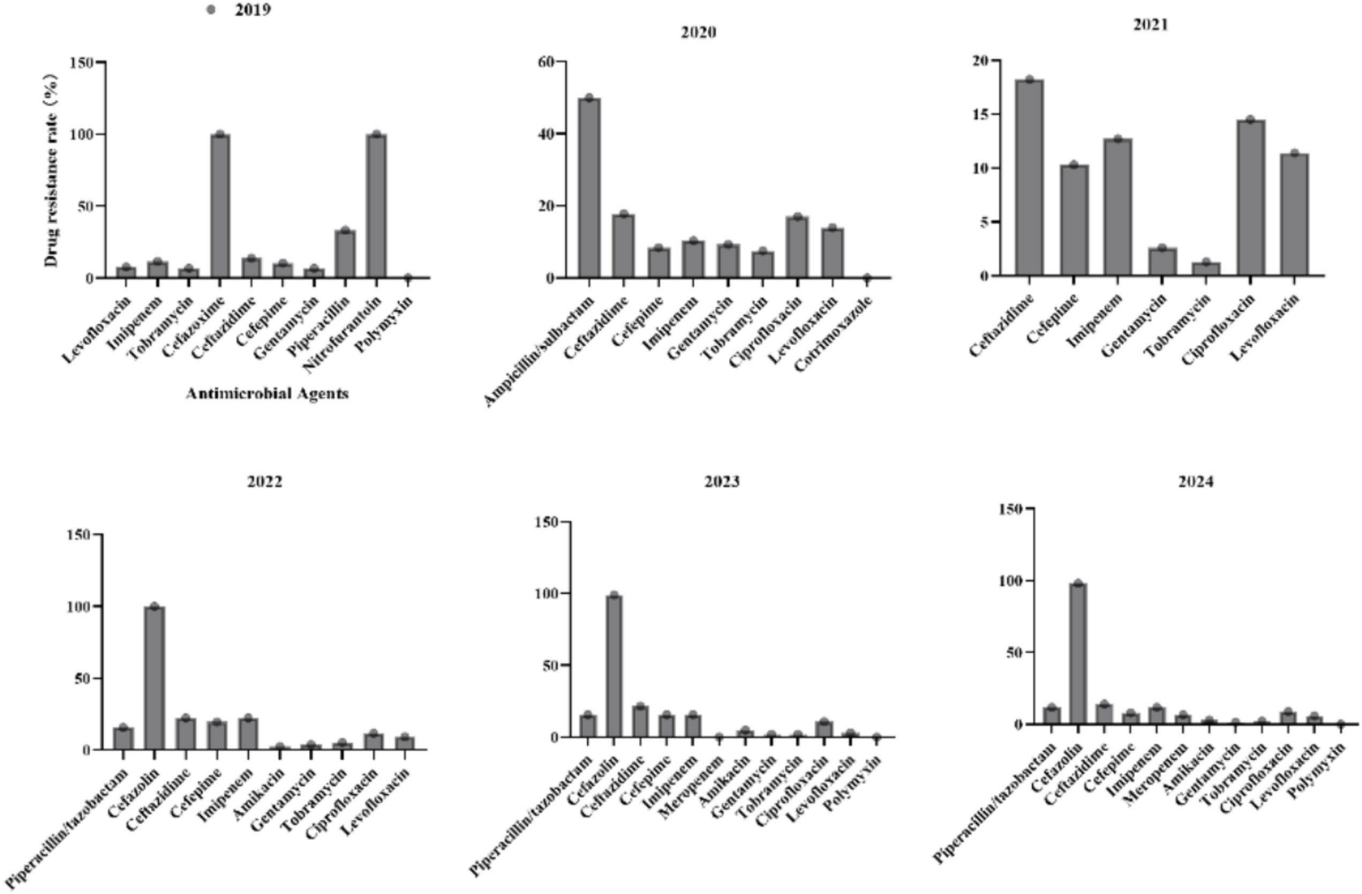
Antimicrobial resistance profiles of *Pseudomonas aeruginosa* to commonly used antibiotics.

For *P. aeruginosa* (Figure 7), the resistance to Ceftazidime initially increased from 13.8% in 2019 to 21.6% in 2023, followed by a significant reduction to 14% in 2024. Resistance to Imipenem exhibited cyclical fluctuations, peaking at 22.4% in 2022 before declining to 11.8% in 2024. Amikacin, Gentamicin, and Tobramycin maintained consistently low resistance rates (<10%) throughout the study period, highlighting their reliability for clinical use. Resistance to Ciprofloxacin and Levofloxacin fluctuated without a clear trend. Polymyxin demonstrated sustained effectiveness with no observed resistance (0%) across all reported years.

### 3.6 Antimicrobial resistance profiles of *Acinetobacter baumannii complex* to commonly used antibiotics from 2019 to 2024

**Figure 8:**
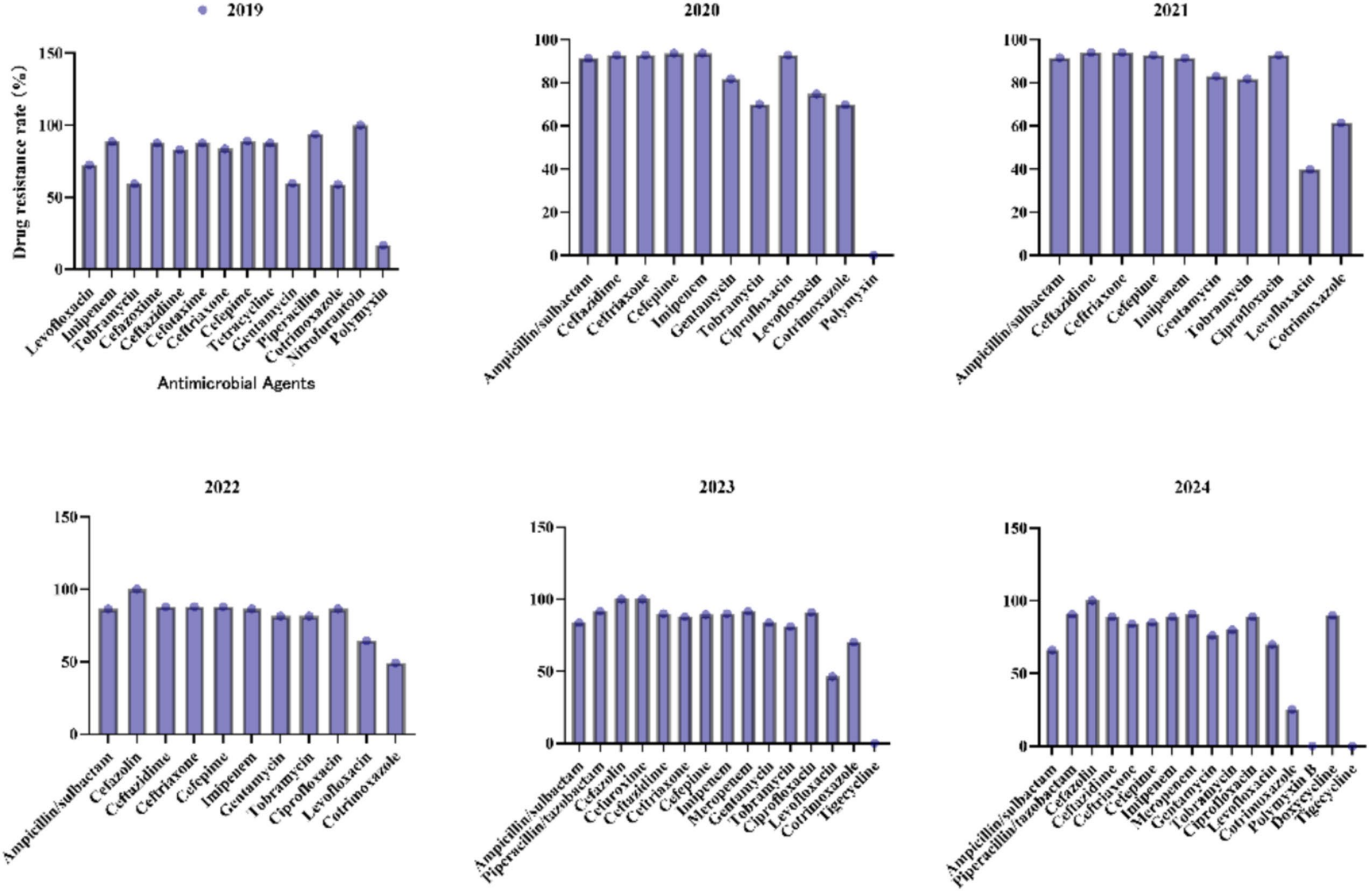
Antimicrobial resistance profiles of *Acinetobacter baumannii complex* to commonly used antibiotics.

For the *A. baumannii complex* (Figure 8), carbapenems such as imipenem and meropenem exhibited consistently high resistance rates (>88%) across all years, with meropenem reaching a peak of 91.7% in 2023. Tobramycin resistance increased steadily from 59.3% in 2019 to 79.9% in 2024. Although resistance to ampicillin/sulbactam has declined from 91.3% in 2020 to 66% in 2024, it remains critically elevated, thereby limiting its clinical utility. Resistance to levofloxacin showed significant fluctuations, decreasing sharply from 72.6% in 2019 to 39.8% in 2021, before increasing again to 69.9% in 2024. Polymyxin B and tigecycline maintained 0% resistance in 2024, underscoring their importance as viable options for treating multidrug-resistant infections.

## 4 Discussion

The intensive care unit (ICU) represents a critical battleground in the global AMR crisis, where the interplay of host vulnerability, invasive interventions, and microbial adaptability fosters the emergence and dissemination of MDR Gram-negative pathogens. This study delineates a concerning epidemiological landscape in western China, characterized by escalating carbapenem resistance in *A. baumannii* (CRAB: 62.14%) and *K. pneumoniae* (CRKP: 29.19%), mirroring global trends of ICU-acquired infections dominated by “ESKAPE” pathogens. The ICU environment—marked by immunosuppression, prolonged device use (e.g., ventilators, catheters), and frequent broad-spectrum antibiotic exposure—creates a permissive niche for MDR colonization and biofilm formation. Notably, bloodstream (45.27%) and respiratory (41.34%) infections predominate, reflecting the vulnerability of critically ill patients to invasive procedures and impaired mucosal barriers, which facilitate pathogen translocation and systemic dissemination (Vincent et al., 2020).

The dominance of *K. pneumoniae* and *A. baumannii*—accounting for over 60% of isolates, the fluctuating prevalence of *A. baumannii complex* 42.23% in 2019 to 26.47 in 2024. The marked decline in *P. aeruginosa* isolates (13.50 to 6.81%) contrasts with the rising prevalence of *K. pneumoniae* (27.01 to 33.63%), mirroring trends observed in Asia and Europe where *K. pneumoniae* has emerged as a dominant nosocomial pathogen due to its adaptability and carbapenemase-producing strains (Nordmann et al., 2012; Tacconelli et al., 2018). The 11.6-fold increase in *H. influenzae* and the emergence of *S. marcescens* (0 to 2.31%) suggest shifts in infection dynamics, possibly linked to changes in antibiotic prophylaxis or host immune status (Schelenz et al., 2016). However, its persistent dominance in respiratory infections (37.85%) highlights the urgent need for enhanced environmental decontamination and stewardship in ICUs. The staggering carbapenem resistance rates in *A. baumannii* (CRAB: 62.14%) and *K. pneumoniae* (CRKP: 29.19%) signal a regional epidemic of carbapenemase-producing strains, likely exacerbated by the overuse of empiric carbapenems in critically ill patients (Cortés et al., 2020). This aligns with national surveillance data from China, where CRAB prevalence in ICUs exceeds 50%, driven by clonal dissemination of blaOXA-23-harboring strains (Jiang et al., 2019; Liu and Liu, 2021). These findings align with global surveillance data, where carbapenemase genes (e.g., blaNDM, blaOXA-48) have disseminated rapidly in healthcare settings (Grundmann et al., 2017; Logan and Weinstein, 2017). The transient decline in CRKP resistance (22% in 2024) may reflect localized stewardship interventions, but the overall trajectory remains concerning. Similarly, the high resistance of *A. baumannii* to meropenem (91.7% in 2023) underscores the limited therapeutic options for these pathogens, necessitating strict adherence to carbapenem-sparing protocols (Tamma et al., 2022). To break the cycle of resistance, a dual focus on antimicrobial stewardship and environmental decontamination is essential. Machine learning models integrating real-time resistance data, patient comorbidities, and antibiotic consumption patterns could optimize empiric therapy (Feretzakis et al., 2020). Additionally, novel non-antibiotic adjuvants—such as phage therapy targeting CRAB biofilms or efflux pump inhibitors to restore carbapenem susceptibility—offer promise in preclinical studies (Strathdee et al., 2023). Locally, the high CRAB prevalence warrants investment in strict contact precautions and UV-C disinfection robots, which reduced CRAB transmission by 67% in a Korean ICU trial (Park et al., 2023).

The observed resistance patterns reveal pathogen-specific evolutionary strategies. *A. baumannii*’s pan-resistance to carbapenems and aminoglycosides correlates with its genomic plasticity, enabling rapid acquisition of resistance islands and efflux pump overexpression (Abd El-Rahman et al., 2023). In contrast, *K. pneumoniae*’s resistance to third-generation cephalosporins (e.g., ceftazidime) and carbapenems may stem from co-carriage of *blaCTX-M-15* and *blaNDM-5* on conjugative plasmids, facilitating horizontal gene transfer (Yang et al., 2023). The lower carbapenem resistance in *E. coli* (5.68%) compared to *K. pneumoniae* suggests ecological partitioning of resistance genes, possibly due to fitness costs associated with carbapenemase production in *E. coli* (Partridge et al., 2018). However, the fluctuating resistance of *E. coli* to cephalosporins highlights dynamic shifts in ESBL epidemiology, potentially linked to antibiotic cycling practices or community-acquired strain introductions (Tamma et al., 2021). The data necessitate a paradigm shift in ICU antimicrobial strategies. First, the high CRAB burden demands restrictive carbapenem use and adoption of rapid molecular diagnostics (e.g., PCR for *blaOXA-51*) to differentiate colonization from true infection, minimizing unnecessary treatment (van Belkum et al., 2019). Second, the rising CRKP prevalence calls for tailored empiric regimens: in regions with >20% CRKP, ceftazidime-avibactam or meropenem-vaborbactam should replace carbapenems as first-line therapy for sepsis, guided by local antibiograms (Tamma et al., 2021).

The *E. coli* maintained low carbapenem resistance (≤1.3%), consistent with its lesser propensity for carbapenemase production compared to Klebsiella and Acinetobacter species. However, the rising resistance to cephalosporins (e.g., ceftriaxone: 43.7%) signals the spread of extended-spectrum β-lactamases (ESBLs), urging the adoption of rapid diagnostics to guide targeted therapy (Paterson and Bonomo, 2005). The decline in fluoroquinolone resistance for *E. coli* (ciprofloxacin: 55.2 to 46.1%) and *K. pneumoniae* (levofloxacin: 43.8 to 26.5%) likely reflects reduced empirical use due to stewardship programs, as evidenced by similar trends in North America (Ramirez and Tolmasky, 2010). In contrast, the erratic resistance patterns of levofloxacin in *A. baumannii* (72.6–69.9%) and *P. aeruginosa* (7.7 to 5.9%) suggest pathogen-specific adaptive mechanisms, such as efflux pump overexpression or target mutations (van Belkum et al., 2019). Aminoglycosides retained moderate efficacy for *P. aeruginosa* (<10% resistance), but rising resistance in *A. baumannii* (tobramycin: 59.3–79.9%) and *K. pneumoniae* (gentamicin: 59.7–76%) highlights the gradual erosion of these agents’ utility. This trend parallels reports from high-resistance regions, where aminoglycoside-modifying enzymes have become widespread (Hooper and Jacoby, 2016).

While this single-center study provides granular insights into regional resistance dynamics, its retrospective design limits causal inference. The absence of genotypic data (e.g., whole-genome sequencing) precludes mapping resistance gene transmission networks. However, the large sample size (7,211 isolates) and longitudinal design (2019–2024) strengthen its validity as a sentinel report on MDR trends in western China.

## 5 Conclusion

The ICU continues to serve as a focal point for the evolution of multidrug-resistant (MDR) pathogens, driven by factors such as microbial resilience, therapeutic necessities, and environmental contamination. Addressing this challenge necessitates the integration of precision diagnostics, antibiotic stewardship programs, and advanced disinfection technologies, marking a paradigm shift from reactive treatment strategies to proactive ecological interventions aimed at disrupting resistance reservoirs.

## Data Availability

The original contributions presented in the study are included in the article/Supplementary material, further inquiries can be directed to the corresponding author.

## References

[ref1] Abd El-Rahman O. A. Rasslan F. Hassan S. S. Ashour H. M. Wasfi R. (2023). The RND efflux pump gene expression in the biofilm formation of *Acinetobacter baumannii*. Antibiotics 12:419. doi: 10.3390/antibiotics12020419, PMID: 36830328 PMC9952185

[ref4] Cortés A. Rooney J. Bartley D. J. Nisbet A. J. Cantacessi C. (2020). Helminths, hosts, and their microbiota: new avenues for managing gastrointestinal helminthiases in ruminants. Expert Rev. Anti-Infect. Ther. 18, 977–985. doi: 10.1080/14787210.2020.1782188, PMID: 32530331

[ref5] Doron S. Davidson L. E. (2013). Antimicrobial stewardship. Mayo Clin. Proc. 86, 1113–1123. doi: 10.4065/mcp.2011.0358 PMC3203003 22033257

[ref6] Dulanto Chiang A. Dekker J. P. (2019). Efflux pump-mediated resistance to new beta lactam antibiotics in multidrug-resistant gram-negative bacteria. Commun. Med. 4, 1–9. doi: 10.1038/s43856-024-00591-y PMC11362173 39210044

[ref7] Feretzakis G. Loupelis E. Sakagianni A. Kalles D. Lada M. Christopoulos C.. (2020). Using machine learning algorithms to predict antimicrobial resistance and assist empirical treatment. Stud. Health Technol. Inform. 272, 75–78. doi: 10.3233/SHTI200497, PMID: 32604604

[ref8] Grundmann H. Glasner C. Albiger B. Aanensen D. M. Tomlinson C. T. Andrasević A. T.. (2017). Occurrence of carbapenemase-producing Klebsiella pneumoniae and *Escherichia coli* in the European survey of carbapenemase-producing Enterobacteriaceae (EuSCAPE): a prospective, multinational study. Lancet Infect. Dis. 17, 153–163. doi: 10.1016/S1473-3099(16)30257-2, PMID: 27866944

[ref9] Hooper D. C. Jacoby G. A. (2016). Topoisomerase inhibitors: fluoroquinolone mechanisms of action and resistance. Cold Spring Harb. Perspect. Med. 6:5320. doi: 10.1101/cshperspect.a025320, PMID: 27449972 PMC5008060

[ref001] ICPIC. (2019). Abstracts from the 5th International Conference on Prevention & Infection Control. Antimicrob Resist Infect Control 8 (Suppl 1), 148. doi: 10.1186/s13756-019-0567-6 PMC4474787 28256991

[ref10] Jiang L. Liang Y. Yao W. Ai J. Wang X. Zhao Z. (2019). Molecular epidemiology and genetic characterisation of carbapenem-resistant *Acinetobacter baumannii* isolates from Guangdong Province, South China. J. Glob. Antimicrob. Resist. 17, 84–89. doi: 10.1016/j.jgar.2018.11.002, PMID: 30445207

[ref11] Jiang H. Pu H. Huang N. (2023). Risk predict model using multi-drug resistant organism infection from neuro-ICU patients: a retrospective cohort study. Sci. Rep. 13:15282. doi: 10.1038/s41598-023-42522-2, PMID: 37714922 PMC10504308

[ref12] Liu B. Liu L. (2021). Molecular epidemiology and mechanisms of Carbapenem-resistant *Acinetobacter baumannii* isolates from ICU and respiratory department patients of a Chinese university hospital. Infect Drug Resist 14, 743–755. doi: 10.2147/IDR.S299540, PMID: 33658811 PMC7920613

[ref13] Logan L. K. Weinstein R. A. (2017). The epidemiology of Carbapenem-resistant Enterobacteriaceae: the impact and evolution of a global menace. J. Infect. Dis. 215, S28–s36. doi: 10.1093/infdis/jiw282, PMID: 28375512 PMC5853342

[ref003] Murray C. J. L. Ikuta K. S. Sharara F. Swetschinski L. Aguilar G. R. Gray A.. (2022). Global burden of bacterial antimicrobial resistance in 2019: a systematic analysis. Lancet 399, 629–655. doi: 10.1016/S0140-6736(21)02724-0 35065702 PMC8841637

[ref14] Nordmann P. Gniadkowski M. Giske C. G. Poirel L. Woodford N. Miriagou V. (2012). Identification and screening of carbapenemase-producing Enterobacteriaceae. Clin. Microbiol. Infect. 18, 432–438. doi: 10.1111/j.1469-0691.2012.03815.x, PMID: 22507110

[ref15] Park S. M. Suh J. W. Ju Y. K. Kim J. Y. Kim S. B. Sohn J. W.. (2023). Molecular and virulence characteristics of carbapenem-resistant *Acinetobacter baumannii* isolates: a prospective cohort study. Sci. Rep. 13:19536. doi: 10.1038/s41598-023-46985-1, PMID: 37945745 PMC10636183

[ref16] Partridge S. R. Kwong S. M. Firth N. Jensen S. O. (2018). Mobile genetic elements associated with antimicrobial resistance. Clin. Microbiol. Rev. 31, e00088–17. doi: 10.1128/CMR.00088-17, PMID: 30068738 PMC6148190

[ref17] Paterson D. L. Bonomo R. A. (2005). Extended-spectrum beta-lactamases: a clinical update. Clin. Microbiol. Rev. 18, 657–686. doi: 10.1128/CMR.18.4.657-686.2005, PMID: 16223952 PMC1265908

[ref18] Ramirez M. S. Tolmasky M. E. (2010). Aminoglycoside modifying enzymes. Drug Resist. Updat. 13, 151–171. doi: 10.1016/j.drup.2010.08.003, PMID: 20833577 PMC2992599

[ref19] Schelenz S. Hagen F. Rhodes J. L. Abdolrasouli A. Chowdhary A. Hall A.. (2016). First hospital outbreak of the globally emerging *Candida auris* in a European hospital. Antimicrob. Resist. Infect. Control 5:35. doi: 10.1186/s13756-016-0132-5, PMID: 27777756 PMC5069812

[ref20] Strathdee S. A. Hatfull G. F. Mutalik V. K. Schooley R. T. (2023). Phage therapy: from biological mechanisms to future directions. Cell 186, 17–31. doi: 10.1016/j.cell.2022.11.017, PMID: 36608652 PMC9827498

[ref21] Tacconelli E. Carrara E. Savoldi A. Harbarth S. Mendelson M. Monnet D. L.. (2018). Discovery, research, and development of new antibiotics: the WHO priority list of antibiotic-resistant bacteria and tuberculosis. Lancet Infect. Dis. 18, 318–327. doi: 10.1016/S1473-3099(17)30753-3, PMID: 29276051

[ref22] Tamma P. D. Aitken S. L. Bonomo R. A. Mathers A. J. Van Duin D. Clancy C. J. (2021). Infectious Diseases Society of America guidance on the treatment of extended-Spectrum β-lactamase producing Enterobacterales (ESBL-E), Carbapenem-resistant Enterobacterales (CRE), and *Pseudomonas aeruginosa* with difficult-to-treat resistance (DTR-*P. aeruginosa*). Clin. Infect. Dis. 72, e169–e183. doi: 10.1093/cid/ciaa1478 33106864

[ref23] Tamma P. D. Aitken S. L. Bonomo R. A. Mathers A. J. Van Duin D. Clancy C. J. (2022). Infectious Diseases Society of America 2022 guidance on the treatment of extended-Spectrum β-lactamase producing Enterobacterales (ESBL-E), Carbapenem-resistant Enterobacterales (CRE), and *Pseudomonas aeruginosa* with difficult-to-treat resistance (DTR-*P. aeruginosa*). Clin. Infect. Dis. 75, 187–212. doi: 10.1093/cid/ciac268, PMID: 35439291 PMC9890506

[ref24] Van Belkum A. Bachmann T. T. Lüdke G. Lisby J. G. Kahlmeter G. Mohess A.. (2019). Developmental roadmap for antimicrobial susceptibility testing systems. Nat. Rev. Microbiol. 17, 51–62. doi: 10.1038/s41579-018-0098-9, PMID: 30333569 PMC7138758

[ref25] Vincent J. L. Sakr Y. Singer M. Martinloeches I. Machado F. R. Marshall J. C.. (2020). Prevalence and outcomes of infection among patients in intensive care units in 2017. *JAMA the*. JAMA J. Am. Med. Assoc. 323:1478. doi: 10.1001/jama.2020.2717, PMID: 32207816 PMC7093816

[ref26] Yang J. T. Zhang L. J. Lu Y. Zhang R. M. Jiang H. X. (2023). Genomic insights into global Bla(CTX-M-55)-positive *Escherichia coli* epidemiology and transmission characteristics. Microbiol Spectr 11:e0108923. doi: 10.1128/spectrum.01089-23, PMID: 37358409 PMC10434037

