# Supplementary material for: Distribution patterns and evolution of antimicrobial resistance in Gram-negative bacteria within the intensive care unit of a tertiary hospital from 2019 to 2024
Source: Front Microbiol. 2025 May 15;16:1587132. doi: 10.3389/fmicb.2025.1587132 (PMC12119562; doi:10.3389/fmicb.2025.1587132)
Supplement: Supplementary file 1 [file Data_Sheet_1.PDF]

## Supplementary material

### 1. Definition of Drug-Resistant Strains

MDR is defined as the resistance to three or more distinct classes of antibiotics simultaneously. Specifically, carbapenem-resistant strains include: *carbapenem-resistant Escherichia coli* (CRECO), *carbapenem-resistant Acinetobacter baumannii* (CRAB), *carbapenem-resistant Klebsiella pneumoniae* (CRKP), and *carbapenem-resistant Pseudomonas aeruginosa* (CRPA). These strains are characterized by their resistance to any of the carbapenem antibiotics, including imipenem, meropenem, or biapenem.
